# Frequency, predictors and outcomes of intradialytic complications in patients on maintenance haemodialysis in Dar es Salaam: Prospective longitudinal study

**DOI:** 10.1371/journal.pone.0300823

**Published:** 2025-03-28

**Authors:** John Robert Dugilo, Fatma Bakshi, Muzdalifat Abeid, Samina Somji

**Affiliations:** 1 Department of Internal Medicine, Aga Khan University Medical College, Dar es Salaam, Tanzania; 2 Department of Internal Medicine, Nephrology, Aga Khan University Medical College, Dar es Salaam, Tanzania; 3 Department of Obstetrics and Gynecology, Aga Khan University Medical College, Dar es Salaam, Tanzania; 4 Department of Internal Medicine, Infectious Disease, Aga Khan University Medical College, Dar es Salaam, Tanzania; Warren Alpert Medical School of Brown University: Brown University Warren Alpert Medical School, UNITED STATES OF AMERICA

## Abstract

**Introduction:**

Hemodialysis is a crucial renal replacement therapy option for end stage renal disease (ESRD) patients. Currently, there is a rise of patients who require hemodialysis with concurrent rise in intradialytic complications which can potentiate several outcomes some of which are life threatening. This study assessed the frequency, predictors, and outcomes of intradialytic complications amongst ESRD patients on maintenance hemodialysis.

**Methodology:**

Prospective longitudinal study using self-designed questionnaires including patient’s demographic data and relevant past medical history, pre-hemodialysis assessment and intra-dialysis monitoring was done for 2 months at Aga Khan Hospital and Muhimbili National Hospital, in Dar es salaam, Tanzania.

**Results:**

215 patients were enrolled, of which 138(64.2%) were males with mean age 57(SD 15.37), height 1.64(SD 0.08) and weight 69.27(SD 12.62). Most patients 197(91.6%), were on thrice weekly schedule of which the duration of each session in most patients 206(95.8%) was 4 hours. Diabetes mellitus was the most common etiology of ESRD 126 (58.6%), ArterioVenous fistula (AVF) was the most common vascular access for the procedure 90(41.9%) and mostly, high flux dialyzers were used, FX100 & FX80, (211, 98.2%). There was a statistically significant association between pre-dialysis vital signs, blood flow rate and sex (p value <  0.05) with intradialytic hypertension and hypotension. Interestingly, male sex appeared to elicit a protective effect on intradialytic hypotension (p value < 0.001).

**Conclusion:**

Hemodialysis is a life-saving procedure with multiple complications of which some have detrimental outcomes. Nonetheless, having a good understanding of the factors associated with the complications, appropriate management and ways of preventing them will remarkably improve the procedure and make it a safer renal replacement modality. Carefully, monitoring pre-dialysis vitals and taking necessary measures when deranged, individualized proper machine settings, sufficient fluid removal and standard blood flow rate may improve the dialysis procedure.

## Introduction

End Stage Renal Disease (ESRD) is defined as a permanent reduction of kidney capacity to the extent of becoming life-threatening without dialysis or transplantation. According to the 2012 KDIGO (Kidney Disease Improving Global Outcomes) clinical practice guideline, chronic kidney disease (CKD) is defined as kidney damage evidenced by elevated Creatinine level or reduced glomerular filtration rate and is classified in five stages (stage 1, stage 2. Stage 3a, stage 3b, stage 4 and stage 5) [1].

End Stage Renal Disease (ESRD) is the ultimate complication of almost any kidney disease with highest morbidity and mortality mostly due to cardiovascular compromise, often resulting from Diabetes mellitus, Hypertension, HIV/AIDS and autoimmune diseases especially when timely intervention has not been provided. [1].

According to WHO, worldwide, 13.4 percent (11.7 – 15.1%) of the population is estimated to have CKD of which approximately 5-10 million people are dying every year and by 2030, 5 million people are expected to have ESRD. Morbidity and mortality are not the only concerns but also economic and social burden [1–3].

Several studies report the prevalence for CKD in Africa to be 15.8 percent which is found to be higher in Sub-Saharan Africa where Tanzania is located and is more in high-risk populations specifically amongst elderly or those with chronic diseases than the rest of the population [4]. As study done by O’Hare AM et al reported prevalence and mortality in CKD is higher in elderly as compared to the young patients [4].

In Tanzania, prevalence lies between 7-15 percent and recently there has been an exponential increase in patients with CKD who have advanced to ESRD needing renal replacement therapy (RRT) modalities [3].

Several factors have been implicated as a cause of CKD of which commonest ones are diabetes, hypertension, infectious, autoimmune, and congenital diseases. Once CKD develops ongoing damage through cellular and molecular response happens which propagates disease to further stages by damaging more nephrons and worsening kidney function [1,4,5].

CKD patients have wide range of clinical presentations from asymptomatic (stages 1-3) to life threatening clinical features and complications (stages 4-5) like hematuria, anemia, electrolytes imbalance, metabolic bone disease, cardiovascular diseases, bleeding disorders, neurological disorders, gastrointestinal disorders, depression, uremia, metabolic acidosis, fluid overload skin manifestations and sudden death.

Several laboratory and imaging studies including kidney biopsy are indicated when there is kidney disease or suspected kidney disease depending on the most likely causes of the CKD.

The mainstay treatment of CKD relies on early diagnosis, treating and preventing predisposing factors and complications. Despite supportive treatment of ESRD, the major therapeutic modalities are the dialysis (hemodialysis being most commonly used than peritoneal dialysis (PD)) and renal transplant.

In our settings, the main cornerstone RRT modality in adults with ESRD is hemodialysis with standard settings of four hours per session twice or thrice weekly depending on several factors including patient and economic status [6,7].

Hemodialysis, despite being an essential treatment for ESRD which improves the quality of life, is associated with several complications ranging from mild to life threatening and hence adds more burdens to the patients over and above the experience of the disease itself, with the potential to result in mortality [6,7].

Intradialytic complication (IDC) is an alteration on baseline clinical or biochemical status during dialysis, which can be acute (happens during or immediately after dialysis session) or chronic and may necessitate intervention: pharmacological, invasive, or non-invasive [7].

Most common intra-dialytic complications encountered in patients undergoing maintenance hemodialysis are blood pressure changes (Hypotension and Hypertension), nausea and vomiting, muscle cramps, headache, and chest pain. Despite an increase in availability of services and improvement of quality, mortality in ESRD has risen exponentially in the past one decade, mostly due to intradialytic complications [1,3,8–10].

Hemodialysis as a crucial therapeutic option for ESRD, most studies has reported several complications whereby hypotension was found to be the leading complication in many studies. According to Ali M, et al study done January 2021, most common intradialytic complications are hypotension (28.1%), hypertension (17.0%), nausea and vomiting (11.75%), fever (8.5%), chest pain (7.4%) and others 3% (fever, muscle cramps) [11]. Raja SM, et al reported the frequency of intradialytic complications to be around 30%whereby hypotension was the most common complications [12]. Halle MP, et al reported intradialytic hypotension occurred in 11.6% of Hemodialysis sessions and the incidence of intradialytic hypertension was 48.3% [13]. Several other studies have shown the same proportions or magnitude of intradialytic complications but with different frequencies due to different background ethnicity, culture and underlying comorbidities but intradialytic hypotension has been shown to be the most common complications in most studies [2,6,7,9,12,14].

According to Raja SM, et al. Study shows association between intradialytic hypertension and DM, UF volume and eating during HD, also found that there was association between use of central line catheter as a vascular access with increase in complications rates, however twice weekly HD sessions had the same spectrum of complications as thrice weekly [12]. Prabhakar, Singh RG, et al found out several factors related to intradialytic complications which can be patient-related (like underlying chronic illness of which cardiovascular diseases was the most common, vascular access, medications, eating during HD) also machine-related factors (ultra filtration rate, dialysate temperature and contents) [15]. Halle MP, et al found that intradialytic hypotension was associated with older age, female sex, HIV infection, feeding during dialysis, and use of antihypertensive drug, also found that intradialytic hypertension was associated with Male sex, blood transfusion, dry weight, UF rate [13].

Bleyer AJ, et al reported 42% of dialysis deaths were documented as sudden or cardiac in origin, with 22% of deaths related to cardiac arrest and arrhythmias [16]. Recurrent complications interfered with the well-being of patients including psychological impacts and added more burdens to the disease itself like admissions.

As such this study has helped to determine the frequency and the predictors of intradialytic complications which can be addressed to avoid future complications from reoccurring ultimately improving the patient’s quality of life by addressing the knowledge gaps and influence practice guidelines and policy changes in our poor resource settings.

## Objectives

### Primary objectives

To determine the rate of occurrence of intradialytic complications amongst ESRD patients on maintenance hemodialysis over a period of two months in a tertiary hospitals in Dar es salaam.

### Secondary objectives

1. To determine the risk of admission due to intradialytic complications amongst ESRD patients on maintenance hemodialysis over a period of two months in Dar es salaam.
2. To determine the predictors of Intradialytic complications in patients on maintenance hemodialysis over a period of two months in Dar es salaam.

## Methods

### Study design

This study adopted a longitudinal study in which participants were recruited prospectively.

### Study variables

Dependent variables were Intradialytic complications and admission due to intradialytic complications; and Independent variables: these are the factors which are associated with intradialytic complications: Age, Sex, Comorbidities (Diabetes mellitus and hypertension), Body Mass Index (BMI), Duration of being on maintenance hemodialysis in months, Frequency of hemodialysis per week, Predialysis evaluation (Predialysis weight, vital signs and dry weight), Vascular access, Intradialytic evaluation (UF, duration of the session, vital signs, dialyzer, BFR, DFR), Machine factors.

### Study site

The study was conducted at Aga Khan Hospital and Muhimbili National Hospital, as these are two largest university and tertiary hospitals with large dialysis centers in the city with more than 400 patients doing hemodialysis weekly giving 800 – 1100 sessions per week throughout study duration and they all adhere to national guidelines of monitoring pre-, intra-, and post- dialysis indicators.

### Study duration

The study period was two months started from on 16^th^ October to 22^nd^ December 2022. This duration allowed for the enrollment of an adequate number of hemodialysis sessions.

### Study population

The study included ESRD patients who were on maintenance hemodialysis for at least three months at AKH and MNH dialysis units throughout the study duration. Inclusion criteria were: Patients with ESRD equal or above 18 years of age on hemodialysis for at least three months and exclusion criteria were: severely ill-patient, patient with sepsis or Acute Kidney Injury (AKI), pregnant women and patient with dementia or disorientation or psychiatric disorders.

### Sample size estimation

The sample size obtained from a formula for logistic regression:

n = 100 + 50i. Whereby = Sample size, i = Dependent variable and 50 = EPV

N = 100 + 50x2

N = 200 considering 10%of attrition rate= 220

### Sampling procedure

Through a convenient non-probability sampling method, 215 patients were recruited, as we were using two dialysis centers with approximately 400 patients of whom 180 dialysis sessions were done daily. The study involved hemodialysis sessions of all the patients who met eligibility criteria.

### Research tools

There was a self-designed questionnaire which comprised three components: Demographic details, previous patient’s medical history and pre-dialysis evaluation part and lastly intra- and post dialytic monitoring. Daily monitoring chart which comprises pre-, intra-, and post-intradialytic data.

### Data collection procedures

Through convenient non-probability sampling techniques all patients who fulfilled inclusion criteria from AKH and MNH were enrolled to meet the sample size calculated above of 220.Following approval from the ethical review board of AKU and MNH, informed consent was obtained from every eligible patient enrolled in the study. On average two dialysis sessions per week per patient for those on thrice weekly schedule and one dialysis session per week per patient for those on twice weekly schedule were recorded from recruited patients for that specified study duration on a questionnaire regarding: daily pre-dialysis assessment and intradialytic monitoring.

Two research assistants were trained in how to collect data. Social-demographic data recorded were Age, sex, race, employment status and duration since hemodialysis initiation. Pre- and intra- dialytic patient factors like symptoms before sessions, vital signs before and during session, medications before and during sessions, heparin use during session and eating during dialysis were recorded but also machine factors were collected; blood flow rate, dialysate flow rate, machine temperature, dialysate use and time for the session.

Throughout the study all participants were using the same type of machine (Fresenius dialysis machine 5008S), same dialysate contents and individualized machine settings within normal limits based on patient`s conditions and clinical evaluations.

All the complications were noted during each dialysis session, evaluation for predictors and outcomes was done also, and any participant who developed IDC was managed according to the national guideline for managing intradialytic complications.

### Data management

The data obtained was cleaned, entered in a storage device, and processed before analysis done using SPSS version 25.0 to ensure accuracy, completeness, and uniformity. Throughout the process confidentiality of the data was ensured and after completion of the study, all data were handed over to AKU Faculty of Health science as per section 4.1.6(f) of the faculty manual of research policies and procedures.

### Data analysis

Statistical analysis was done using IBM Statistical Package for the Social Sciences (SPSS) Statistics for Windows, version 25. Sociodemographic data were analyzed and presented as mean with standard deviations for age, Monthly hemoglobin, weight, height, and duration of hemodialysis, while frequency and percentages were calculated for all the categorical data. Stratification was done according to sex, race, employment status, dialysis access, dialyzer, BMI and sessions per week. Association between predictors and occurrence of intradialytic complications will be assessed using Generalizing Estimating Equation (GEE) and expressed as odds ratio.

### Ethical considerations

Ethical approval for conducting research was sought from the research committee of AKU-Dar and written permission was taken from AKH and MNH regarding collecting data and using their facility. Also, all patients gave informed consent before starting to collect information from them and all the information obtained throughout the study including the findings were returned back to the patient. All patients who develop intradialytic complications were managed according to the standard national guideline of managing IDC.

## Results

### Enrollment of the participants

**Fig 1:**
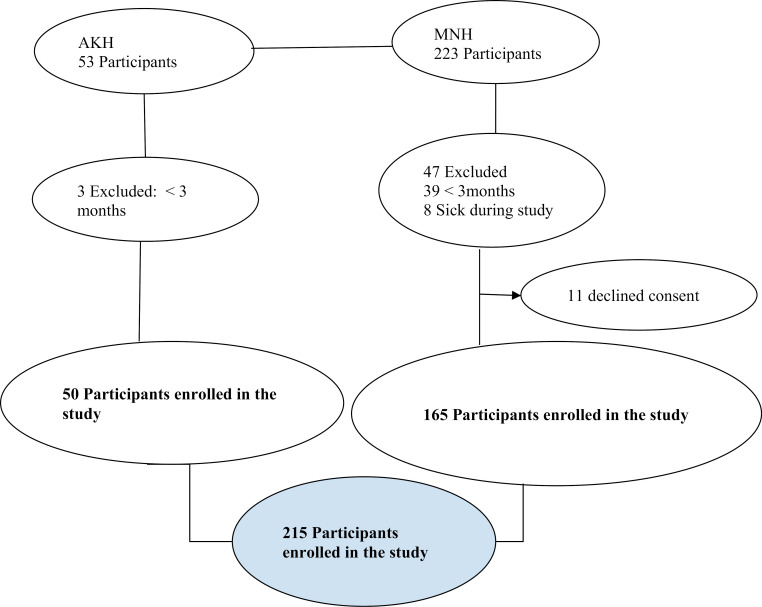
Enrolment of the participants from two tertiary hospitals.

215 participants enrolled, 50 from AKH and 165 from MNH (Fig 1).

#### Descriptive statistics.

215 participants who had ESRD on maintenance hemodialysis were enrolled in the study of which 138 (64.2%) and 77 (35.8%) were males and females respectively with a mean duration of hemodialysis 29 ± 23.23 months, mean age of 57 ± 15.37 years, mean weight 69.27 ± 12.62, mean height 1.64 ± 0.08M and mean monthly hemoglobin 10.72 ± 1.65.

Majority of the participants were on thrice weekly scheduling 197(91.6%) of which the duration of each session in most patients was 4 hours 206(95.8%). Over a period of two months, 2111 sessions were carried out, whereby 1970 and 141 were in the thrice-weekly and twice-weekly scheme respectively. Most of the participants were found to be on hemodialysis between one and five years (55.8%, n = 120), ArterioVenous fistula (AVF) was the most vascular access for the procedure 90(41.9%) and mostly, high flux dialyzers were used, FX100 & FX80, (211, 98.2%). Diabetes mellitus was the most common etiology of ESRD 126(58.6%) followed by Hypertension 77(35.5) Table 1. 

**Table 1:** Baseline characteristics of esrd patients on maintenance hemodialysis followed for 2 months in Dar es Salaam.

Number of patients (215)	
**Total dialysis sessions (2111)**	
**Variables**	**Frequency**
**Sex (n, %)**	
Male	138(64.2)
Female	77(35.8)
**Age**	
Young adults (18-39)	31(14.4)
Middle-aged adults (40-59)	92(42.8)
Old adults (60-100)	92(42.8)
**Duration of hemodialysis**	
Less than one year	72(35.5)
1-5 years	120(55.8)
More than 5 years	23(10.7)
**BMI (n, %)**	
Less than 18.5(underweight)	7(3.3)
18.5 - 24.9 (Normal weight)	89(41.4)
25 - 29 (Overweight)	87(40.5)
30 or high (Obese)	32(14.8)
**Duration of hemodialysis per session (n, %)**	
3 hours	5(2.3)
3.5 hours	4(1.9)
4 hours	206(95.8)
**Frequency of hemodialysis (n, %)**	
Twice weekly	18(8.4)
Thrice weekly	197(91.6)
**Comorbidities (n, %)**	
Diabetes mellitus	126(58.6)
Hypertension	77(35.5)
HIV	37(17.2)
Hepatitis B infection	3(1.4)
Hepatitis C infection	5(2.8)
Others	61(28.4)
**Vascular access (n, %)**	
Temporary catheter	86(40)
Permanent catheter	39(18.1)
AVF	90(41.9)

AVF =  ArterioVenous Fistula, BMI =  Body Mass Index, HIV = Human Immunodeficiency Virus, n = Number, % =  Percentage

The study found hypertension was the most common intradialytic complication, 560 episodes of intradialytic hypertension with incidence rate of 26.5 per 100 dialysis sessions, followed by Intradialytic hypotension 156 episodes with incidence rate of 7.4 per 100 sessions and Fever 25 episodes with incidence rate of 1.2 per 100 sessions. The overall incidence of developing intradialytic complications was 36.6%.

From Table 2; above explains all sessions (2111) which were comprised by visit per participant whereby visit one to seven had all 215 participants but eight to ten as indicated in the table (n), all the complications and it`s incidence were listed for each visit in column, rows give the total number of complications and it`s incidence across the all visits [Table 3].

**Table 2:** Distribution of intradialytic complications amongst ESRD patients on maintenance hemodialysis followed for 2 months in Dares Salaam.

Number of patients (n) = 215												
Total number of hemodialysis sessions = 2111												
Total number of intradialytic complications observed = 770												
Type of intradialytic complication	Dialysis sessions											
	1 n = 215	2 n = 215	3 n = 215	4 n = 215	5 n = 215	6 n = 215	7 n = 215	8 n = 210	9 n = 208	10 n = 188	Total dialysis sessions n = 2111	**I**ncidence of intradialytic complications (per 100 session interval
Intradialytic hypertension (n, %)	54 (73)	48 (69.6)	63 (85.1)	69 67.6	58 (69)	53 (74.6)	58 (72.5)	54 (67.5)	53 (74.6)	50 (76.9)	560 (72.7)	26.5
Intradialytic hypotension (n, %)	10 (13.5)	13 (18.8)	6 (8.1)	26 (25.5)	19 (22.6)	15 (21.2)	14 (17.5)	24 (30)	16 (22.5)	13 (20)	156 (20)	7.4
Fever (n, %)	5 (6.8)	3 (4.3)	1 (1.4)	3 (2.9)	3 (3.6)	1 (1.4)	4 (5)	1 (1.3)	2 (2.9)	2 (3.1)	25 (3.3)	1.2
Muscle cramps (n. %)	3 (4)	4 (5.8)	1 (1.4)	3 (2.9)	3 (3.6)	1 (1.4)	3 (3.7)	1 (1.3)	0	0	19 (2.6)	1
Nausea and Vomiting (n, %)	2 (2.7)	1 (1.5)	2 (2.7)	1 (1)	0	0	1 (1.3)	0	0	0	7 (1)	0.3
Bleeding (n, %)	0	0	1 (1.4)	0	1 (1.2)	1 (1.4)	0	0	0	0	3 (0.4)	0.1
Total number of complications per session.	74 (100)	69 (100)	74 (100)	102 (100)	84 (100)	71 (100)	80 (100)	80 (100)	71 (100)	65 (100)	770 (100)	36.5

**Table 3:** Distribution of hospital admissions due to intradialytic complications amongst ESRD patients on maintenance hemodialysis followed for 2 months.

Number of ESRD patients, n (215)			
**Total number of dialysis sessions, N (2111)**			
**Total number of admissions from any cause observes 53(24.7%)**			
**Total number of admissions due to intradialytic complications observed 15 (7%)**			
**Session**	**Number of admissions due to intradialytic complications**	**Number of ESRD patients**	**Risk of admission amongst ESRD patients (%)**
1	2	215	0.9
2	2	215	0.9
3	1	215	0.5
4	1	215	0.5
5	2	215	0.9
6	2	215	0.9
7	0	215	0
8	2	210	1
9	1	208	0.5
10	2	188	1.1
Total	**15**		**7.2**

#### Inferential statistics.

Among the objectives of this study was to detect predictors of intradialytic complications, which was achieved by Generalizing Estimating Equation as shown in **Table 4**. According to the findings shown in the table there was a statistically significant association between pre-dialysis vital signs, blood flow rate, sex (p value less than 0.05) with occurrence of intradialytic hypertensive events. Having abnormal vitals, specifically high blood pressure before dialysis predisposes ESRD patients to developing intradialytic hypertension while blood flow rate has a protective effect on occurrence of intradialytic hypertension. Notably age, comorbidities, pre dialysis weight, dry weight and UF were not significantly associated with occurrence of intradialytic hypertensive events (p value more than 0.05).

**Table 4:** Predictors of occurrence of intradialytic hypertensive events in ESRD patients on maintenance hemodialysis for two months.

Number of patients 215			
Total number of hemodialysis sessions 2111			
Total number of intradialytic hypertensive episodes 560 (26.5%)			
Variable	P value	Adjusted OD	95% CI
Age	0.298	1.011	0.990–1.033
Sex			
Male	0.001	0.047	0.008–0.283
Female	0.002	0.040	0.005-0.300
Comorbidities			
Diabetes mellitus	0.457	0.795	0.435–1.454
Hypertension	0.087	0.276	0.063–1.208
Duration hemodialysis in months	0.827	0.998	0.984-1.013
Monthly hemoglobin	0.920	0.992	0.844–1.165
Pre-dialysis evaluation			
Frequency of hemodialysis per week	0.829	0.883	0.286–2.731
Duration of hemodialysis per session	0.020	9.425	1.434–61.932
Pre-dialysis vital signs	<0.001	9.578	5.387–17.029
Intra-dialysis vital signs	<0.001	3.172	1.371–7.335
Pre-dialysis weight	0.902	1	0.996–1.004
Dry weight	0.747	1.004	0.979–1.030
Intradialytic evaluation			
Ultra filtration (Ltr)	0.582	1.082	0.818–1.430
Blood flow rate (BFR)	<0.001	0.987	0.980–0.994
Dialysate flow rate (DFR)	0.055	1.001	1.000–1.002
Intradialytic eating	0.297	0.673	0.320–1.415
Intradialytic medications	0.980	1.025	0.140–7.536

According to the table below there was a statistically significant association between sex, pre-dialysis vital signs and blood flow rate (p value less than 0.001) with intradialytic hypotension. Having abnormal vitals, specifically low blood pressure before dialysis predisposes ESRD patients to develop intradialytic hypotension while normal blood flow rate and being male had a protective effect on occurrence of intradialytic hypotension. Notably, comorbidities, pre dialysis weight, dry weight and UF were not significantly associated with occurrence of intradialytic hypotension (p value more than 0.05) [Table 5].

**Table 5:** Predictors of occurrence of intradialytic hypotensive events in ESRD patients on maintenance hemodialysis for two months.

Number of patients 215			
Total number of hemodialysis sessions 2111			
Total number of intradialytic hypotensive episode 156 (7.4%)			
Variable	P value	Adjusted OD	95% CI
Age	0.558	1.006	0.986 – 1.027
Sex			
Male	0.045	0.016	0 -0.910
Female	0.052	0.014	0 – 1.034
Comorbidities			
Diabetes mellitus	0.484	0.809	0.448 - 1.464
Hypertension	0.181	0.393	0.100 – 1.543
Duration hemodialysis in months	0.291	0.993	0.981 – 1.006
Monthly hemoglobin	0.444	0.939	0.799 – 1.104
Pre-dialysis evaluation			
Frequency of hemodialysis per week	0.887	1.074	0.401 – 2.876
Duration of hemodialysis per session	0.142	2.228	0.354 – 1.418
Pre-dialysis vital signs	<0.001	5.723	3.193 – 10.257
Intra-dialysis vital signs	<0.001	0.004	0.001 – 0.011
Pre-dialysis weight	0.745	1	0.995 – 1.003
Dry weight	0.561	1.007	0.984 – 1.030
Intradialytic evaluation			
Ultrafiltration (Ltr)	0.503	1.074	0.872 – 1.322
Blood flow rate (BFR)	0.001	0.989	0.983 – 0.996
Dialysate flow rate (DFR)	0.008	1.001	1.000 – 1.002
Intradialytic eating	0.355	0.712	0.346 – 1.462
Intradialytic medications	0.679	1.411	0.276 – 7.204

## Discussion

Hemodialysis is the preferred type of Renal Replacement Therapy modality used worldwide with remarkable evolution in its safety and easy to use, though cost-effective and life-saving modality it can predispose to significant life-threatening complications despite considerable improvement in the machines and procedure protocols.

Most of the End stage renal disease patients are at considerable risk of developing intradialytic complications due to the underlying chronic illnesses which compromise immunity, cardiac and hemodynamic stability [17].

The main objective of this study is to determine the rate of occurrence of intradialytic complications amongst ESRD patients on maintenance hemodialysis over a period of two months in tertiary hospitals.

### Rate of occurrence of intradialytic complications

Hypertension is the leading intradialytic complication (26.5%. n =  560) as seen in this study, multiple factors are postulated as the mechanism but the most common ones are interdialytic weight gain, inadequate fluid removal and poorly controlled chronic hypertension. Intradialytic hypertension increases cardiovascular complications rates, hospitalizations and mortality. Contrary to our study most studies reported intradialytic hypertension as second common complications after hypotension, Islam et al. [7], Ali M [11], Mehmood Y [18]. In this study, intradialytic hypertension is leading because poor follow up of dry weight hence inadequate fluid removal, poorly controlled chronic hypertension which manifested by high blood pressure before dialysis and inappropriate use of anti-hypertensives.

Hypotension is the second most common intradialytic complications (7.4%, n = 156) after hypertension observed in the study; significant weight gain, excessive ultrafiltration and pre-dialysis medications are among the most postulated mechanisms for intradialytic hypotension [17,19]. Most studies reported intradialytic hypotension as the most common complication contrary to our study Islam et al reported 12.62% [6,7,17,20,21]. The reason for low frequency of intradialytic hypotension in our study is the advancement of machines which can detect slight drop of blood of blood pressure before becoming symptomatic hence necessary intervention can be taken to prevent further drop and therefore increase knowledge to prevent hypotension during hemodialysis.

Fever was the third most complication to occur in this study (1.2%, n =  25) and the most common attributed causes was catheter related infection or underlying sepsis or bacteremia though it was low as compared to other studies Ali m et al reported 8%. Mahamood et al 1.4% [11,17].

Nausea and vomiting was seen only in 0.3% much lower to most of the studies and the reason being advancement in the standard setting of the machine as is one of the implicated common cause and intradialytic hypotension Shaikh RA et al reported 3.22% [5,19].

Muscle cramps reported were seen in only 1% which was postulated mostly from excessive fluid removal or electrolytes imbalances, bleeding seen in only 0.1% and was due to catheter related bleeding.

Other intradialytic complications found in our study less than 0.1%, headache, chest pain and hemorrhagic stroke, Mehmood Y reported 50% experienced headaches which associated with longer dialysis sessions and excessive fluid removal [18], Ali M reported 7.4% experienced chest pain [11], Mahamood et al found 15% experienced chest pain [17] and Islam et al found 1.47% postulated mechanisms for chest pain during hemodialysis mostly attributed due to low hemoglobin level [7].

### Risk of admission due to intradialytic complications

In our study, the risk of admission due to intradialytic complications among End stage renal disease was found to be 7% which was attributed by intradialytic bleeding due to heparin toxicity, catheter-related sepsis, neurological and cardiological compromise, catheter related thrombosis and severe intradialytic hypertension or hypotension failed to resolve during dialysis treatment or after termination of treatment, Inrig JK,et al reported significant mortality and admissions in patient who undergoing maintenance hemodialysis within 2 years [6].

### Predictors of intradialytic complications

**Patient factors:** We focused on were: age, sex, chronic diseases (comorbidities), duration of hemodialysis since initiation, monthly hemoglobin, weight (pre-dialysis weight and dry weight), blood pressure (pre dialysis session blood pressure), intradialytic eating and medications.

Of all the predictors mentioned above study found there was no difference between male and female for intradialytic complications contrary to the findings of Halle MP et al. [13] whereby in that study there was association between male sex with intradialytic hypertension and female sex with intradialytic hypotension whereby this study didn`t find such association.

Also having high blood pressure before dialysis predispose to develop intradialytic hypertension while in intradialytic hypotension, no such observed association in the study.

For other factors, the study found no significant statistical association contrary to study done by Raja SM et al. [12], Halle MP [13].

**Procedure factors** considered were: Frequency of dialysis per week, duration of hemodialysis per session and amount of fluid to be removed per session (UF).There were no associations between the above factors and intradialytic hypertension/hypotension in the study contrary to the study done by Halle MP et al. [13] found association between UF and intradialytic hypertension.

Machine factors: Throughout the study all the participants used the same kind of dialysis machine with most of the settings being the same but we focused on variable machine settings; blood flow rate and dialysate flow, whereby study found there was significant statistical association between intradialytic hypotension and hypertension with the above mentioned factors.

### Strength and limitations

#### Strength.

1. First longitudinal study on intradialytic complications to be done in the country whereby it gives some basis or insights of further studies to be done.
2. It gives more insight on the factors related to intradialytic complications especially in the area which might be influenced by many sociocultural and financial factors.

#### Limitations.

1. As longitudinal study more study duration is required
2. To involve more dialysis centers in the country as diversity of factors could be easily captured.
3. Involvement of biochemical and cardiac profiles as it might contribute to the occurrence of intradialytic complications

## Conclusion

Hemodialysis is a life-saving procedure with multiple complications of which some have detrimental outcomes. Nonetheless, having a good understanding of the factors associated with the complications, appropriate management and ways of preventing them will remarkably improve the procedure and make it safer renal replacement modality.

From the study, carefully monitoring of pre-dialysis vitals specifically high or low blood pressure that can propagate complications if no necessary measures taken when deranged, individualized proper machine settings for preventing machine related complications, sufficient fluid removal based on frequent dry weight assessment and standard blood and dialysate flow rate will predict and even pre-empting complications hence improve dialysis procedure.

### Recommendations

Long duration longitudinal study involving more variables including biochemical and cardiovascular profiles is recommended

All predictors weather patient, procedure or machine factors implicated as a cause of intradialytic complications mentioned in the study should be properly evaluated before the initiation of dialysis for screening hence preventing the complication to occur

## Supporting information

S1 File Data set for study: Intradialytic complications-modified. (XLSX)
