# Supplementary material for: Frequency, predictors and outcomes of intradialytic complications in patients on maintenance haemodialysis in Dar es Salaam: Prospective longitudinal study
Source: PLoS One. 2025 Mar 28;20(3):e0300823. doi: 10.1371/journal.pone.0300823 (PMC11952240; doi:10.1371/journal.pone.0300823)
Supplement: Attachment — Submitted filename: John Dugilo rebuttal letter for PLOS ONE_073731.docx [file pone.0300823.s004.docx]

To the editor and reviewers,

Title of Manuscript: **Frequency, Predictors and Outcomes of Intradialytic Complications in patients on maintenance haemodialysis in Dar es salaam: Prospective longitudinal study.**

**Thank you very much for taking your time to review our work and for constructive feedback. Below is a list of our response.**

Author: John Dugilo

| **S/No.** | **EE comments** | **Action taken** |
| --- | --- | --- |
| 1. | Please ensure that your manuscript meets PLOS ONE's style requirements, including those for file naming | Changes made to adhere to the requirements. |
| 2. | Please amend either the title on the online submission form (via Edit Submission) or the title in the manuscript so that they are identical. | The title in the manuscript has been amended to resemble title on the online submission form. |
| 3. | Please include your full ethics statement in the ‘Methods’ section of your manuscript file. In your statement, please include the full name of the IRB or ethics committee who approved or waived your study, as well as whether or not you obtained informed written or verbal consent. If consent was waived for your study, please include this information in your statement as well. | Full ethics statement has been included in the methods section. |
| 4. | We note you have included a table to which you do not refer in the text of your manuscript. Please ensure that you refer to Table 1,3 and 5 in your text; if accepted, production will need this reference to link the reader to the Table. | Tables 1, 3 and 5 have been referred to the text of manuscript. |
| 5. | The authors tend to use very long sentences, which obscures the intended meaning of the text. I have highlighted some of these in the article (attached) but would suggest proof-reading from a native English speaker. | Proof reading done and edits made accordingly as highlighted: A2, A5, A6, A7, A8, A9, A10, A11, A12, A13, A14, A18, A19, A20, A21, A29, A30, A31. |
| 6. | In the Methods, some comment about the self-designed questionnaires would be useful. What informed the use of the parameters used? A literature review on similar questionnaires used to determine intradialytic complications would have been useful. (A16) | An amendment has been made to explain the source of the parameters used in the questionnaire. |
| 7. | Define the intradialytic complications: for example intradialytic hypertension and hypotension. There are several definitions used particularly for intradialytic hypotension. Could your rather low incidence be due to the fact that your definition was different from previous studies? (A22, A24) | Intradialytic hypertension and hypotension are clearly defined in the manuscript as per the definitions outlined in the proposal. These consistent definitions were employed throughout the research and in drawing our conclusions. |
| 8. | Not all the dialysis sessions were recorded. Was there a reason for picking some of the dialysis sessions, instead of recording all? Each dialysis session is an event in itself so it would be justifiable to record all sessions. If the authors have a valid reason for not doing so, it should be stated in the methods (A17) | Clarification has been provided on why not all dialysis sessions were recorded and how this approach still aligned with the study goals. |
| 9. | Average UF and blood flow rates would be useful to understand the practices of the dialysis centers. What blood flow rate? High or low? What are the values? (A23, A25) | Elaboration has been added to the manuscript text. |
| 10. | Please attach the self-designed questionnaire for completeness. | Self-designed questionnaire has been attached. |
| 11. | The references should conform to PLOS one style. (A1, A3 & A4) | The referencing style has been amended to conform to PLOS one guidelines. |
| 12. | Perhaps weeks may be better here for more precision. This period comes to 9 weeks and 4 days (A15) | Two months has been changed to nine weeks. |
| 13. | A26: This seems like a repetition of the introduction. Your discussion should focus on the results of your study. | Amendment has been done to focus on the study results. |
| 14. | A27: How often do you time your machines to determine blood pressure, or is a blood pressure drop automatically detected? Please describe this process with more detail. | The machine automatically detects the drop of blood pressure below the normal cut-off range. |
| 15. | Although rare, this is a serious complication and deserves more description. E.g hemorrhagic stroke occurred in one individual who.. | Has been well elaborated in the manuscript text. |
| 16. | The investigation reports a series of intradialytic complications (hypo-hypertension, bleeding, etc.) already well-defined in the literature. | We conducted this investigation in our specific settings to understand local variations and challenges. |
| 17. | The description of these events remains undetailed regarding the patients, such as the cardiac parameters for hypotension or the dialytic technique, such as the value of Na in the dialysate. | The lack of detailed descriptions of events was due to limited availability of the cardiac evaluation resources however for dialysis technique; we use 5008S machines for all patients, maintaining consistent dialysate contents with individualized settings tailored to each patient`s needs. |
| 18. | The study is limited to 2 months, which is not enough time to assess the problem of intradialytic tolerance. The number of patients was not numerous as well. | The study`s was limited to two months due to financial constraints. The number of participants was determined based on a formula and a review of the literature to ensure adequate representation within our resource limits. |
| 19. | The terms primary and secondary objectives should be replaced with primary and secondary endpoints. | Changed as recommended |
| 20. | The point of risk of admission should be clarified even though it is intuitive | Has been clarified in the manuscript text of table 3 |
| 21. | In the Discussion, the sentence “The reason for low…..during hemodialysis “should be rephrased because the possibility of the instruments catching the early drop of blood pressure doesn’t delete, for the investigation, that an adverse event occurred. | We record all drops of blood pressure which were symptomatic only. |
