# Supplementary material for: Frequency, predictors and outcomes of intradialytic complications in patients on maintenance haemodialysis in Dar es Salaam: Prospective longitudinal study
Source: PLoS One. 2025 Mar 28;20(3):e0300823. doi: 10.1371/journal.pone.0300823 (PMC11952240; doi:10.1371/journal.pone.0300823)
Supplement: Attachment — Submitted filename: Rebuttal letter.docx [file pone.0300823.s005.docx]

To the editor and reviewers,

Title of Manuscript: **Frequency, Predictors and Outcomes of Intradialytic Complications in patients on maintenance haemodialysis in Dar es salaam: Prospective longitudinal study.**

**Thank you very much for taking your time to review our work and for constructive feedback. Below is a list of our response.**

Author:John Dugilo

| **S/No.** | **EE comments** | **Action taken** |
| --- | --- | --- |
| 1. | The introduction is unnecessarily lengthy. Consider removing background information about established causes, presentation, and investigations, focusing instead on treatment approaches and local practice context. | The changes have been made as per the recommendations. |
| 2. | Capitalize 'S' after "et al." in references | The change has been done as advised. |
| 3. | Standardize abbreviations: Define "IDC" (intradialytic complication) at first use and maintain consistency throughout | Intradialytic complication (IDC) refer to alteration in a patient`s baseline clinical or biochemical status during dialysis. These complications can be acute – occurring during or immediately after dialysis session - or chronic, and they may require pharmacological, invasive, or non-invasive interventions |
| 4. | Revise verb tense in methodology sections (e.g., change "will be assessed" to "was assessed" in data analysis) | Verb tense has been revised |
| 5. | Table 4 and 5 formatting requires adjustment. Recommend reorganizing columns as: Odds Ratio \| 95% CI \| p-value | Tables have been formatted |
| 6. | The following statement requires clarification: "Most studies reported intradialytic hypotension as the most common complication contrary to our study. Islam et al reported..." | Clarification has been provided: While most studies reported intradialytic hypotension as the most common complication, our study found a lower frequency. For instance Islam et al reported a prevalence of 12.62%^(Islam et al., 2017)^, Mahamood et al ^(Mahmood et al., 2018),(Inrig et al., 2007; Sars, van der Sande, & Kooman, 2020)^ also observed higher rates |
| 7. | The manuscript would benefit from thorough proofreading and language editing. These revisions would strengthen what is otherwise a solid contribution to the field. | Language editing and proofreading have been completed. |
