# Supplementary material for: Frequency, predictors and outcomes of intradialytic complications in patients on maintenance haemodialysis in Dar es Salaam: Prospective longitudinal study
Source: PLoS One. 2025 Mar 28;20(3):e0300823. doi: 10.1371/journal.pone.0300823 (PMC11952240; doi:10.1371/journal.pone.0300823)
Supplement: Peer review history

# Author comment: Author response to Decision Letter 0

**DOI:** 10.1371/journal.pone.0300823.r001

5 Mar 2024

# Peer review: Decision Letter 0

**DOI:** 10.1371/journal.pone.0300823.r002

**Authors:** Ankur Shah

29 Apr 2024

PONE-D-24-06139FREQUENCY, PREDICTORS AND OUTCOMES OF INTRADIALYTIC COMPLICATIONS IN PATIENTS ON MAINTENANCE HAEMODIALYSIS IN DAR ES SALAAM:  PROSPECTIVE LONGITUDINAL STUDY.PLOS ONE

Dear Dr. Dugilo,

Thank you for submitting your manuscript to PLOS ONE. After careful consideration, we feel that it has merit but does not fully meet PLOS ONE’s publication criteria as it currently stands. Therefore, we invite you to submit a revised version of the manuscript that addresses the points raised during the review process.

Please submit your revised manuscript by Jun 13 2024 11:59PM. If you will need more time than this to complete your revisions, please reply to this message or contact the journal office at plosone@plos.org . When you're ready to submit your revision, log on to https://www.editorialmanager.com/pone/ and select the 'Submissions Needing Revision' folder to locate your manuscript file.

Please include the following items when submitting your revised manuscript:

- A rebuttal letter that responds to each point raised by the academic editor and reviewer(s). You should upload this letter as a separate file labeled 'Response to Reviewers'.
- A marked-up copy of your manuscript that highlights changes made to the original version. You should upload this as a separate file labeled 'Revised Manuscript with Track Changes'.
- An unmarked version of your revised paper without tracked changes. You should upload this as a separate file labeled 'Manuscript'.

If you would like to make changes to your financial disclosure, please include your updated statement in your cover letter. Guidelines for resubmitting your figure files are available below the reviewer comments at the end of this letter.

If applicable, we recommend that you deposit your laboratory protocols in protocols.io to enhance the reproducibility of your results. Protocols.io assigns your protocol its own identifier (DOI) so that it can be cited independently in the future. For instructions see: https://journals.plos.org/plosone/s/submission-guidelines#loc-laboratory-protocols . Additionally, PLOS ONE offers an option for publishing peer-reviewed Lab Protocol articles, which describe protocols hosted on protocols.io. Read more information on sharing protocols at https://plos.org/protocols?utm_medium=editorial-email&utm_source=authorletters&utm_campaign=protocols .

We look forward to receiving your revised manuscript.

Kind regards,

Ankur Shah

Academic Editor

PLOS ONE

Journal Requirements:

When submitting your revision, we need you to address these additional requirements.

1. Please ensure that your manuscript meets PLOS ONE's style requirements, including those for file naming. The PLOS ONE style templates can be found at 

https://journals.plos.org/plosone/s/file?id=wjVg/PLOSOne_formatting_sample_main_body.pdf and 

https://journals.plos.org/plosone/s/file?id=ba62/PLOSOne_formatting_sample_title_authors_affiliations.pdf

2. Please amend either the title on the online submission form (via Edit Submission) or the title in the manuscript so that they are identical.

3. Please include your full ethics statement in the ‘Methods’ section of your manuscript file. In your statement, please include the full name of the IRB or ethics committee who approved or waived your study, as well as whether or not you obtained informed written or verbal consent. If consent was waived for your study, please include this information in your statement as well. 

4. We note you have included a table to which you do not refer in the text of your manuscript. Please ensure that you refer to Table 1,3 and 5 in your text; if accepted, production will need this reference to link the reader to the Table.

**Additional Editor Comments:**

Thank you for your submission, please address the reviewers comments.

[Note: HTML markup is below. Please do not edit.]

Reviewers' comments:

Reviewer's Responses to Questions

**Comments to the Author**

1. Is the manuscript technically sound, and do the data support the conclusions?

The manuscript must describe a technically sound piece of scientific research with data that supports the conclusions. Experiments must have been conducted rigorously, with appropriate controls, replication, and sample sizes. The conclusions must be drawn appropriately based on the data presented. 

Reviewer #1: Partly

Reviewer #2: Partly

**********

2. Has the statistical analysis been performed appropriately and rigorously? 

Reviewer #1: I Don't Know

Reviewer #2: Yes

**********

3. Have the authors made all data underlying the findings in their manuscript fully available?

The PLOS Data policy requires authors to make all data underlying the findings described in their manuscript fully available without restriction, with rare exception (please refer to the Data Availability Statement in the manuscript PDF file). The data should be provided as part of the manuscript or its supporting information, or deposited to a public repository. For example, in addition to summary statistics, the data points behind means, medians and variance measures should be available. If there are restrictions on publicly sharing data—e.g. participant privacy or use of data from a third party—those must be specified.

Reviewer #1: No

Reviewer #2: Yes

**********

4. Is the manuscript presented in an intelligible fashion and written in standard English?

PLOS ONE does not copyedit accepted manuscripts, so the language in submitted articles must be clear, correct, and unambiguous. Any typographical or grammatical errors should be corrected at revision, so please note any specific errors here.

Reviewer #1: No

Reviewer #2: Yes

**********

5. Review Comments to the Author

Please use the space provided to explain your answers to the questions above. You may also include additional comments for the author, including concerns about dual publication, research ethics, or publication ethics. (Please upload your review as an attachment if it exceeds 20,000 characters)

Reviewer #1: The article describes the incidence and predictors of intradialytic complications amongst end stage renal disease patients in 2 tertiary hospitals in Dar es Salaam, Tanzania. While this is an important study, there are several flaws in the article that need to be addressed:

1. The authors tend to use very long sentences, which obscures the intended meaning of the text. I have highlighted some of these in the article (attached) but would suggest proof-reading from a native English speaker.

2. In the Methods, some comment about the self-designed questionnaires would be useful. What informed the use of the parameters used? A literature review on similar questionnaires used to determine intradialytic complications would have been useful.

3. Define the intradialytic complications: for example intradialytic hypertension and hypotension. There are several definitions used particularly for intradialytic hypotension. Could your rather low incidence be due to the fact that your definition was different from previous studies?

4. Not all the dialysis sessions were recorded. As stated by the authors,: "On average two dialysis sessions per week per patient for those on thrice weekly schedule and one dialysis session per week per patient for those on twice weekly schedule were recorded from recruited patients for that specified study duration on a questionnaire regarding: daily pre-dialysis assessment and intradialytic monitoring". Was there a reason for picking some of the dialysis sessions, instead of recording all? Each dialysis session is an event in itself so it would be justifiable to record all sessions. If the authors have a valid reason for not doing so, it should be stated in the methods.

5. Average UF and blood flow rates would be useful to understand the practices of the dialysis centers. For instance the authors state: "According to the findings shown in the table there was a statistically significant association between pre-dialysis vital signs, blood flow rate........while blood flow rate has a protective effect on occurrence of intradialytic hypertension" What blood flow rate? High or low? what are the values?

6. Please attach the self-designed questionnaire for completeness.

7. The references should conform to PLOS one style.

There are other clarifications needed which I have outlined in the text.

In Summary, the article has merit but the methodology has to be very clear in other to justify the results.

Reviewer #2: This manuscript reports an investigation of intradialytic complications in a cohort of 215 chronic hemodialysed patients. Moreover, the study investigated frequency, possible predictor markers, and outcomes associated with these events. The conclusions state that hemodialysis is a life-saving procedure, but it is still associated with a burden of l multiple complications causing unfortunate outcomes.

A noticeable note is the high prevalence of diabetes as the most frequent cause of renal failure.

The manuscript has some significant drawbacks.

The investigation reports a series of intradialytic complications (hypo-hypertension, bleeding, etc.) already well-defined in the literature.

The description of these events remains undetailed regarding the patients, such as the cardiac parameters for hypotension or the dialytic technique, such as the value of Na in the dialysate.

The study is limited to 2 months, which is not enough time to assess the problem of intradialytic tolerance. The number of patients was not numerous as well.

The terms primary and secondary objectives should be replaced with primary and secondary

endpoints

The point of risk of admission should be clarified even though it is intuitive.

In the Discussion, the sentence “The reason for low…..during hemodialysis “should be rephrased because the possibility of the instruments catching the early drop of blood pressure doesn’t delete, for the investigation, that an adverse event occurred.

**********

6. PLOS authors have the option to publish the peer review history of their article (what does this mean? ). If published, this will include your full peer review and any attached files.

If you choose “no”, your identity will remain anonymous but your review may still be made public.

**Do you want your identity to be public for this peer review?** For information about this choice, including consent withdrawal, please see our Privacy Policy .

Reviewer #1: **Yes: ** Ngozi Virginia Aikpokpo

Reviewer #2: No

**********

[NOTE: If reviewer comments were submitted as an attachment file, they will be attached to this email and accessible via the submission site. Please log into your account, locate the manuscript record, and check for the action link "View Attachments". If this link does not appear, there are no attachment files.]

While revising your submission, please upload your figure files to the Preflight Analysis and Conversion Engine (PACE) digital diagnostic tool, https://pacev2.apexcovantage.com/ . PACE helps ensure that figures meet PLOS requirements. To use PACE, you must first register as a user. Registration is free. Then, login and navigate to the UPLOAD tab, where you will find detailed instructions on how to use the tool. If you encounter any issues or have any questions when using PACE, please email PLOS at figures@plos.org . Please note that Supporting Information files do not need this step.

Attachment Submitted filename: John Dugilo Manuscript_080438.docx

# Author comment: Author response to Decision Letter 1

**DOI:** 10.1371/journal.pone.0300823.r003

2 Sep 2024

1. Please ensure that your manuscript meets PLOS ONE's style requirements, including those for file naming:

Changes made to adhere to the requirements.

2.Please amend either the title on the online submission form (via Edit Submission) or the title in the manuscript so that they are identical:

The title in the manuscript has been amended to resemble title on the online submission form.

3. Please include your full ethics statement in the ‘Methods’ section of your manuscript file. In your statement, please include the full name of the IRB or ethics committee who approved or waived your study, as well as whether or not you obtained informed written or verbal consent. If consent was waived for your study, please include this information in your statement as well:

Full ethics statement has been included in the methods section.

4. We note you have included a table to which you do not refer in the text of your manuscript. Please ensure that you refer to Table 1,3 and 5 in your text; if accepted, production will need this reference to link the reader to the Table:

Tables 1, 3 and 5 have been referred to the text of manuscript.

5. The authors tend to use very long sentences, which obscures the intended meaning of the text. I have highlighted some of these in the article (attached) but would suggest proof-reading from a native English speaker:

Proof reading done and edits made accordingly as highlighted: A2, A5, A6, A7, A8, A9, A10, A11, A12, A13, A14, A18, A19, A20, A21, A29, A30, A31.

6. In the Methods, some comment about the self-designed questionnaires would be useful. What informed the use of the parameters used? A literature review on similar questionnaires used to determine intradialytic complications would have been useful. (A16):

An amendment has been made to explain the source of the parameters used in the questionnaire.

7. Define the intradialytic complications: for example intradialytic hypertension and hypotension. There are several definitions used particularly for intradialytic hypotension. Could your rather low incidence be due to the fact that your definition was different from previous studies? (A22, A24):

Intradialytic hypertension and hypotension are clearly defined in the manuscript as per the definitions outlined in the proposal. These consistent definitions were employed throughout the research and in drawing our conclusions.

8. Not all the dialysis sessions were recorded. Was there a reason for picking some of the dialysis sessions, instead of recording all? Each dialysis session is an event in itself so it would be justifiable to record all sessions. If the authors have a valid reason for not doing so, it should be stated in the methods (A17):

Clarification has been provided on why not all dialysis sessions were recorded and how this approach still aligned with the study goals.

9. Average UF and blood flow rates would be useful to understand the practices of the dialysis centers. What blood flow rate? High or low? What are the values? (A23, A25):

Elaboration has been added to the manuscript text.

10. Please attach the self-designed questionnaire for completeness:

Self-designed questionnaire has been attached.

11. The references should conform to PLOS one style. (A1, A3 & A4):

The referencing style has been amended to conform to PLOS one guidelines.

12. Perhaps weeks may be better here for more precision. This period comes to 9 weeks and 4 days (A15):

Two months has been changed to nine weeks.

13. A26: This seems like a repetition of the introduction. Your discussion should focus on the results of your study:

Amendment has been done to focus on the study results.

14. A27: How often do you time your machines to determine blood pressure, or is a blood pressure drop automatically detected? Please describe this process with more detail:

The machine automatically detects the drop of blood pressure below the normal cut-off range.

15. Although rare, this is a serious complication and deserves more description. E.g hemorrhagic stroke occurred in one individual who:

Has been well elaborated in the manuscript text.

16. The investigation reports a series of intradialytic complications (hypo-hypertension, bleeding, etc.) already well-defined in the literature:

We conducted this investigation in our specific settings to understand local variations and challenges.

17. The description of these events remains undetailed regarding the patients, such as the cardiac parameters for hypotension or the dialytic technique, such as the value of Na in the dialysate:

The lack of detailed descriptions of events was due to limited availability of the cardiac evaluation resources however for dialysis technique; we use 5008S machines for all patients, maintaining consistent dialysate contents with individualized settings tailored to each patient`s needs.

18. The study is limited to 2 months, which is not enough time to assess the problem of intradialytic tolerance. The number of patients was not numerous as well:

The study`s was limited to two months due to financial constraints. The number of participants was determined based on a formula and a review of the literature to ensure adequate representation within our resource limits.

19. The terms primary and secondary objectives should be replaced with primary and secondary

endpoints:

Changed as recommended

20. The point of risk of admission should be clarified even though it is intuitive:

Has been clarified in the manuscript text of table 3

21. In the Discussion, the sentence “The reason for low…..during hemodialysis “should be rephrased because the possibility of the instruments catching the early drop of blood pressure doesn’t delete, for the investigation, that an adverse event occurred:

We record all drops of blood pressure which were symptomatic only.

Attachment Submitted filename: John Dugilo rebuttal letter for PLOS ONE_073731.docx

# Peer review: Decision Letter 1

**DOI:** 10.1371/journal.pone.0300823.r004

**Authors:** Ankur Shah

30 Dec 2024

PONE-D-24-06139R1FREQUENCY, PREDICTORS AND OUTCOMES OF INTRADIALYTIC COMPLICATIONS IN PATIENTS ON MAINTENANCE HAEMODIALYSIS IN DAR ES SALAAM:  PROSPECTIVE LONGITUDINAL STUDY.PLOS ONE

Dear Dr. Dugilo,

Thank you for submitting your manuscript to PLOS ONE. After careful consideration, we feel that it has merit but does not fully meet PLOS ONE’s publication criteria as it currently stands. Therefore, we invite you to submit a revised version of the manuscript that addresses the points raised during the review process.

==============================

**ACADEMIC EDITOR: The authors were responsive to the reviewers comments, additional areas for improvement have been identified - thank you.**==============================

Please submit your revised manuscript by Feb 13 2025 11:59PM. If you will need more time than this to complete your revisions, please reply to this message or contact the journal office at plosone@plos.org . When you're ready to submit your revision, log on to https://www.editorialmanager.com/pone/ and select the 'Submissions Needing Revision' folder to locate your manuscript file.

Please include the following items when submitting your revised manuscript:

- A rebuttal letter that responds to each point raised by the academic editor and reviewer(s). You should upload this letter as a separate file labeled 'Response to Reviewers'.
- A marked-up copy of your manuscript that highlights changes made to the original version. You should upload this as a separate file labeled 'Revised Manuscript with Track Changes'.
- An unmarked version of your revised paper without tracked changes. You should upload this as a separate file labeled 'Manuscript'.

If you would like to make changes to your financial disclosure, please include your updated statement in your cover letter. Guidelines for resubmitting your figure files are available below the reviewer comments at the end of this letter.

If applicable, we recommend that you deposit your laboratory protocols in protocols.io to enhance the reproducibility of your results. Protocols.io assigns your protocol its own identifier (DOI) so that it can be cited independently in the future. For instructions see: https://journals.plos.org/plosone/s/submission-guidelines#loc-laboratory-protocols . Additionally, PLOS ONE offers an option for publishing peer-reviewed Lab Protocol articles, which describe protocols hosted on protocols.io. Read more information on sharing protocols at https://plos.org/protocols?utm_medium=editorial-email&utm_source=authorletters&utm_campaign=protocols .

We look forward to receiving your revised manuscript.

Kind regards,

Ankur Shah

Academic Editor

PLOS ONE

Journal Requirements:

Please review your reference list to ensure that it is complete and correct. If you have cited papers that have been retracted, please include the rationale for doing so in the manuscript text, or remove these references and replace them with relevant current references. Any changes to the reference list should be mentioned in the rebuttal letter that accompanies your revised manuscript. If you need to cite a retracted article, indicate the article’s retracted status in the References list and also include a citation and full reference for the retraction notice.

[Note: HTML markup is below. Please do not edit.]

Reviewers' comments:

Reviewer's Responses to Questions

**Comments to the Author**

1. If the authors have adequately addressed your comments raised in a previous round of review and you feel that this manuscript is now acceptable for publication, you may indicate that here to bypass the “Comments to the Author” section, enter your conflict of interest statement in the “Confidential to Editor” section, and submit your "Accept" recommendation.

Reviewer #3: (No Response)

**********

2. Is the manuscript technically sound, and do the data support the conclusions?

The manuscript must describe a technically sound piece of scientific research with data that supports the conclusions. Experiments must have been conducted rigorously, with appropriate controls, replication, and sample sizes. The conclusions must be drawn appropriately based on the data presented. 

Reviewer #3: Yes

**********

3. Has the statistical analysis been performed appropriately and rigorously? 

Reviewer #3: Yes

**********

4. Have the authors made all data underlying the findings in their manuscript fully available?

The PLOS Data policy requires authors to make all data underlying the findings described in their manuscript fully available without restriction, with rare exception (please refer to the Data Availability Statement in the manuscript PDF file). The data should be provided as part of the manuscript or its supporting information, or deposited to a public repository. For example, in addition to summary statistics, the data points behind means, medians and variance measures should be available. If there are restrictions on publicly sharing data—e.g. participant privacy or use of data from a third party—those must be specified.

Reviewer #3: Yes

**********

5. Is the manuscript presented in an intelligible fashion and written in standard English?

PLOS ONE does not copyedit accepted manuscripts, so the language in submitted articles must be clear, correct, and unambiguous. Any typographical or grammatical errors should be corrected at revision, so please note any specific errors here.

Reviewer #3: No

**********

6. Review Comments to the Author

Please use the space provided to explain your answers to the questions above. You may also include additional comments for the author, including concerns about dual publication, research ethics, or publication ethics. (Please upload your review as an attachment if it exceeds 20,000 characters)

Reviewer #3: (No Response)

The authors have done a good job in assessing the local practice patterns of hemodialysis with an aim to identify predictors of its associated complications to help improve patients life. I would suggest following changes to help the reader understand this manuscript well.

Methodological Comments:

Introduction

- The introduction is unnecessarily lengthy. Consider removing background information about established causes, presentation, and investigations, focusing instead on treatment approaches and local practice context.
- Technical corrections needed:
  Capitalize 'S' after "et al." in references
  Standardize abbreviations: Define "IDC" (intradialytic complication) at first use and maintain consistency throughout
  Revise verb tense in methodology sections (e.g., change "will be assessed" to "was assessed" in data analysis)

Statistical Analysis

- Table 4 and 5 formatting requires adjustment. Recommend reorganizing columns as: Odds Ratio | 95% CI | p-value

Discussion

- The following statement requires clarification: "Most studies reported intradialytic hypotension as the most common complication contrary to our study. Islam et al reported..."

Overall Assessment: While the study's findings align with existing literature, the authors have provided valuable insights into local practice patterns, complication rates, and their associated factors. The manuscript would benefit from thorough proofreading and language editing. These revisions would strengthen what is otherwise a solid contribution to the field.

**********

7. PLOS authors have the option to publish the peer review history of their article (what does this mean? ). If published, this will include your full peer review and any attached files.

If you choose “no”, your identity will remain anonymous but your review may still be made public.

**Do you want your identity to be public for this peer review?** For information about this choice, including consent withdrawal, please see our Privacy Policy .

Reviewer #3: No

**********

[NOTE: If reviewer comments were submitted as an attachment file, they will be attached to this email and accessible via the submission site. Please log into your account, locate the manuscript record, and check for the action link "View Attachments". If this link does not appear, there are no attachment files.]

While revising your submission, please upload your figure files to the Preflight Analysis and Conversion Engine (PACE) digital diagnostic tool, https://pacev2.apexcovantage.com/ . PACE helps ensure that figures meet PLOS requirements. To use PACE, you must first register as a user. Registration is free. Then, login and navigate to the UPLOAD tab, where you will find detailed instructions on how to use the tool. If you encounter any issues or have any questions when using PACE, please email PLOS at figures@plos.org . Please note that Supporting Information files do not need this step.

# Author comment: Author response to Decision Letter 2

**DOI:** 10.1371/journal.pone.0300823.r005

13 Feb 2025

1. The introduction is unnecessarily lengthy. Consider removing background information about established causes, presentation, and investigations, focusing instead on treatment approaches and local practice context.

The changes have been made as per the recommendations.

2. Capitalize 'S' after "et al." in references: The change has been done as advised.

3. Standardize abbreviations: Define "IDC" (intradialytic complication) at first use and maintain consistency throughout: Intradialytic complication (IDC) refer to alteration in a patient`s baseline clinical or biochemical status during dialysis. These complications can be acute – occurring during or immediately after dialysis session - or chronic, and they may require pharmacological, invasive, or non-invasive interventions

4. Revise verb tense in methodology sections (e.g., change "will be assessed" to "was assessed" in data analysis): Verb tense has been revised.

5. Table 4 and 5 formatting requires adjustment. Recommend reorganizing columns as: Odds Ratio | 95% CI | p-value: Tables have been formatted

6. The following statement requires clarification: "Most studies reported intradialytic hypotension as the most common complication contrary to our study. Islam et al reported...": Clarification has been provided: While most studies reported intradialytic hypotension as the most common complication, our study found a lower frequency. For instance Islam et al reported a prevalence of 12.62%(Islam et al., 2017), Mahamood et al (Mahmood et al., 2018),(Inrig et al., 2007; Sars, van der Sande, & Kooman, 2020) also observed higher rates

7. The manuscript would benefit from thorough proofreading and language editing. These revisions would strengthen what is otherwise a solid contribution to the field: Language editing and proofreading have been completed.

Attachment Submitted filename: Rebuttal letter.docx

# Editor report: Decision Letter 2

**DOI:** 10.1371/journal.pone.0300823.r006

**Authors:** Ankur Shah

19 Feb 2025

FREQUENCY, PREDICTORS AND OUTCOMES OF INTRADIALYTIC COMPLICATIONS IN PATIENTS ON MAINTENANCE HAEMODIALYSIS IN DAR ES SALAAM:  PROSPECTIVE LONGITUDINAL STUDY.

PONE-D-24-06139R2

Dear Dr. Dugilo,

We’re pleased to inform you that your manuscript has been judged scientifically suitable for publication and will be formally accepted for publication once it meets all outstanding technical requirements.

Within one week, you’ll receive an e-mail detailing the required amendments. When these have been addressed, you’ll receive a formal acceptance letter and your manuscript will be scheduled for publication.

An invoice will be generated when your article is formally accepted. Please note, if your institution has a publishing partnership with PLOS and your article meets the relevant criteria, all or part of your publication costs will be covered. Please make sure your user information is up-to-date by logging into Editorial Manager at Editorial Manager®  and clicking the ‘Update My Information' link at the top of the page. If you have any questions relating to publication charges, please contact our Author Billing department directly at authorbilling@plos.org.

If your institution or institutions have a press office, please notify them about your upcoming paper to help maximize its impact. If they’ll be preparing press materials, please inform our press team as soon as possible -- no later than 48 hours after receiving the formal acceptance. Your manuscript will remain under strict press embargo until 2 pm Eastern Time on the date of publication. For more information, please contact onepress@plos.org.

Kind regards,

Ankur Shah

Academic Editor

PLOS ONE

Additional Editor Comments (optional):

The authors were responsive to the additional reviewer and the manuscript is suitable for publication.

Reviewers' comments:

# Editor report: Acceptance letter

**DOI:** 10.1371/journal.pone.0300823.r007

**Authors:** Ankur Shah

PONE-D-24-06139R2

PLOS ONE

Dear Dr. Dugilo,

I'm pleased to inform you that your manuscript has been deemed suitable for publication in PLOS ONE. Congratulations! Your manuscript is now being handed over to our production team.

At this stage, our production department will prepare your paper for publication. This includes ensuring the following:

* All references, tables, and figures are properly cited

* All relevant supporting information is included in the manuscript submission,

* There are no issues that prevent the paper from being properly typeset

If revisions are needed, the production department will contact you directly to resolve them. If no revisions are needed, you will receive an email when the publication date has been set. At this time, we do not offer pre-publication proofs to authors during production of the accepted work. Please keep in mind that we are working through a large volume of accepted articles, so please give us a few weeks to review your paper and let you know the next and final steps.

Lastly, if your institution or institutions have a press office, please let them know about your upcoming paper now to help maximize its impact. If they'll be preparing press materials, please inform our press team within the next 48 hours. Your manuscript will remain under strict press embargo until 2 pm Eastern Time on the date of publication. For more information, please contact onepress@plos.org.

If we can help with anything else, please email us at customercare@plos.org.

Thank you for submitting your work to PLOS ONE and supporting open access.

Kind regards,

PLOS ONE Editorial Office Staff

on behalf of

Dr. Ankur Shah

Academic Editor

PLOS ONE
